# Incidence of childhood CNS tumours

**DOI:** 10.1038/sj.bjc.6990502

**Published:** 1999-06

**Authors:** B J Morland, S E Parkes

**Affiliations:** Department of Oncology, Birmingham Children's Hospital NHS Trust, Birmingham; West Midlands Regional Children's Tumour Research Group, Birmingham Children's Hospital NHS Trust, Birmingham, UK


*British Journal of Cancer* (1999) **80**(8), 1301
© 1999 Cancer Research Campaign
Article no. bjoc.1998.0502

Letter to the Editor

### Incidence of childhood CNS tumours

**Sir**

McKinney et al (1998) (*Br J Cancer* **78**: 974–979) recently reported their 22-year regional series of 455 cases of childhood central nervous system (CNS) tumours, in which they demonstrated a consistently increasing annual incidence and an apparent shift in the age distribution of astrocytomas. Geographical analysis revealed a definite distribution pattern at county level, implying a link with large-scale environmental factors, although no definite associations were found with socioeconomic group, ethnicity or population density.

They commented on the paucity of similar reports on the descriptive epidemiology of childhood CNS tumours, but omitted to refer to the study from the West Midlands (Stevens et al, 1991). The West Midlands Regional Children's Tumour Research Group (WMRCTRG) is also a specialist childhood cancer registry, holding population-based data from 1957 onwards on all children resident or treated in the West Midlands, one of the largest Health Authorities in England and Wales. Routine histopathological review has been carried out on over 2000 cases of solid tumour (Parkes et al, 1997), including over 80% of the CNS tumours in the Register.

In our 1980–1984 study of 147 brain tumours (Stevens et al, 1991), we reported an incidence of 26.5 per million children per year, which compares with the 28 in McKinney's study. We have subsequently analysed a further 17 years' worth of data (1975–1996), a total of 697 cases, which demonstrate an overall age-standardized incidence rate for the series of 29.8. As reported in Yorkshire, we have also seen a small significant ($P$ = 0.04) increase in the incidence of all CNS tumour types over the 22-year period from 24 to 29.7 per million per year. However, we observed a large increase in the incidence of astrocytoma over the period ($P$ = 0.002), the only one of the five subtypes to show a significant increase in the West Midlands. Interestingly, we also demonstrated a significant increase in the 0–4-year age-group ($P$ = 0.0004). This supports the age distribution shift reported by McKinney et al, which may reflect improving diagnostic services over time.

McKinney et al also reported a statistically significant increase in the incidence of PNET/medulloblastoma, which agrees with findings in the North Western region, but conflicts with those of Thorne and colleagues (Thorne et al, 1994) who found a statistically significant decline in these tumours in the Southwest region of England. We have previously analysed the incidence in the West Midlands over a similar period (Morland and Parkes, 1995) and demonstrated a non-significant decrease, as was also found in the northern region. The existence of these five regional variations is interesting, although the reason for the differences is as yet unclear.

Geographical analysis has been performed on a subset of the West Midlands data (unpublished report), which suggested that there might be higher rates in the rural areas of the region, but owing to small numbers, this cannot be stated with certainty. It is intended to extend the analysis to a longer time period, which may reveal further evidence.

In summary, it is difficult to explain the contrasting findings and wide variations in incidence of particular subtypes between the regions, which may be accounted for by geographical and environmental influences. It would therefore be interesting to investigate the situation on a national basis with data from all regions, in order to plot the larger topographical picture, which would enable further aetiological hypotheses.

*BJ Morland*
*Department of Oncology, Birmingham Children's Hospital NHS Trust, Birmingham*

*SE Parkes*
*West Midlands Regional Children's Tumour Research Group, Birmingham Children's Hospital NHS Trust, Birmingham, UK*

### REFERENCES

McKinney et al (1998) Epidemiology of childhood brain tumours in Yorkshire, UK, 1974–1995: geographical distribution and changing incidence. *Br J Cancer* **78**: 974–979

Morland BJ and Parkes SE (1995) Response to 'Decline in incidence of medulloblastoma in children'. *Cancer* **76**: 155–156

Parkes SE, Muir KR, Cameron AH, Raafat F, Stevens MCG, Morland BJ, Barber PC, Carey MP, Fox H, Jones EL, Marsden HB, Pincott JR, Pringle JAS, Reid H, Rushton DI, Starkie CM, Whitwell HL, Wright DH and Mann JR (1997) The need for specialist review of pathology in paediatric cancer. *Br J Cancer* **75**: 1156–1159

Stevens MCG, Cameron AH, Muir KR, Parkes SE, Reid H and Whitwell H (1991) Descriptive epidemiology of primary central nervous system tumours in children: a population-based study. *Clin Oncol* **3**: 323–329

Thorne RN, Pearson ADJ, Nicoll JAR, Coakham HB, Oakhill A, Mott MG and Foreman NK (1994) Decline in incidence of medulloblastoma in children. *Cancer* **74**: 3240–3244

